# Decoding Inflammation in Hidradenitis Suppurativa: Serum Cytokines as Biomarkers of Disease Severity and Treatment Outcome—Systematic Review

**DOI:** 10.3390/jcm15124699

**Published:** 2026-06-17

**Authors:** Anna Tekielak, Karina Polak, Aleksandra Frątczak, Beata Bergler-Czop

**Affiliations:** 1 Doctoral School, Medical University of Silesia, 40-055 Katowice, Poland; 2 Department of Dermatology, Medical University of Silesia, Francuska 20/42, 40-027 Katowice, Poland; m.carrine@gmail.com (K.P.); ola.fratczak89@gmail.com (A.F.);

**Keywords:** hidradenitis suppurativa, cytokines, serum biomarkers, biologic therapy

## Abstract

**Introduction**: Hidradenitis suppurativa (HS) is a chronic inflammatory skin disease with systemic features, characterized by painful nodules, abscesses, and sinus tracts. Pro-inflammatory cytokines may serve as biomarkers of disease severity, inflammatory burden, and therapeutic response. The aim of this article is to systematically review the current evidence on serum concentrations of pro-inflammatory cytokines in patients with HS and to evaluate their potential utility as biomarkers of disease activity and personalized treatment guidance. **Materials and Methods**: This systematic review was conducted in accordance with the PRISMA 2020 guidelines. A literature search was performed in PubMed, Embase, and Scopus, with Google Scholar used as a supplementary source. The search included English-language publications from 2015 to 2026. The following keywords were used in combination: “cytokines”, “serum”, “hidradenitis suppurativa”, and “markers”. In addition, reference lists and citations of eligible full-text articles were manually screened. Eligible studies for inclusion were peer-reviewed, original human studies reporting serum cytokine levels in patients with HS. Reviews, meta-analyses, case reports, editorials, letters, in vitro and animal studies, conference abstracts without the full text available, non-serum studies, and thematically unrelated publications were excluded. **Results**: Database searches identified records in PubMed, Embase, Scopus, and Google Scholar, of which three PubMed studies met the inclusion criteria. Manual screening of reference lists and citations identified three additional eligible publications. Overall, six studies were included in the final qualitative synthesis. The included studies reported elevated serum levels of key inflammatory mediators, including IL-17A, IL-6, IL-1β, IL-23, IL-18, and soluble TNF receptors, in patients with HS compared to healthy controls. Several cytokines were associated with disease severity, and selected markers showed changes during biologic treatment. Stratification into immunological endotypes based on cytokine profiles and clinical features was also proposed. **Conclusions**: Pro-inflammatory cytokines, especially those involved in the IL-1β–IL-17 axis, show potential as biomarkers of disease severity and treatment response in HS. Their assessment may support future personalized therapeutic strategies. However, current evidence remains limited by small study numbers and methodological heterogeneity. Further large-scale prospective and longitudinal studies are required before serum cytokine profiling can be implemented in routine clinical practice.

## 1. Introduction

Hidradenitis suppurativa (HS) is a chronic recurrent inflammatory dermatosis characterized by the formation of painful nodules, abscesses, sinus tracts, and fibrotic scarring, predominantly affecting regions rich in apocrine glands, including the axillary, inguinal, anogenital, and gluteal areas, as well as submammary regions. Growing evidence indicates that hidradenitis suppurativa is not only a localized follicular disorder but also a systemic inflammatory disease characterized by altered serum cytokine profiles, involving IL-17 and IL-1 family mediators in particular, as well as selected acute-phase inflammatory markers [1,2,3]. This condition substantially diminishes patients’ quality of life, impacting both their physical and psychological well-being, and is frequently associated with a spectrum of comorbidities, such as obesity, metabolic syndrome, inflammatory bowel disease, depression, and other immune-mediated disorders [3,4].

The pathogenesis of HS remains intricate and only partially elucidated; however, accumulating evidence implicates dysregulation of the follicular–sebaceous–apocrine unit and aberrant immune responses as central pathogenic mechanisms. A prevailing hypothesis suggests that follicular hyperkeratosis and subsequent occlusion precipitate follicular rupture, thereby triggering a cascade of inflammatory events involving both innate and adaptive immune cells [3]. This pathological process is further exacerbated by excessive production of pro-inflammatory cytokines [1,5]. In recent years, serum cytokine profiling has emerged as a promising avenue for identifying disease biomarkers indicative of HS activity and severity. Among the most extensively investigated inflammatory mediators are IL-1β, IL-17, IL-23, interferon-gamma (IFN-γ), and tumor necrosis factor-alpha (TNF-α) [1,6,7,8,9,10,11,12]. Empirical studies reveal that patients with active HS exhibit elevated serum concentrations of these cytokines relative to healthy controls, suggesting a systemic inflammatory component underlying disease pathology. Moreover, cytokine expression patterns in HS appear to be modulated by variables such as ethnicity, body mass index, disease severity, and smoking status [8]. A more comprehensive understanding of the regulatory mechanisms governing serum cytokine levels and their correlation with clinical HS phenotypes could facilitate the development of precision-targeted therapeutic interventions aimed at modulating specific inflammatory pathways.

The aim of this systematic review was to present the current state of knowledge on the serum concentrations of pro-inflammatory cytokines in patients with HS and their potential importance as biomarkers of disease severity and response to biologic therapy. The analysis aimed to assess the relationship between the inflammatory profile and the clinical phenotype of patients, and to identify cytokines that can support the personalization of treatment in daily clinical practice. Despite growing interest in inflammatory biomarkers in HS, the clinical applicability of serum cytokine profiling remains limited and not yet standardized. One of the major challenges is the heterogeneity of published data, resulting from differences in study populations, disease severity, comorbidities, and laboratory methodologies. Moreover, while numerous studies confirm elevated cytokine levels in HS patients, their specificity and sensitivity as reliable biomarkers for clinical decision-making remain unclear. Importantly, the concept of systemic inflammation in HS has gained increasing recognition, suggesting that HS should be regarded not only as a dermatological condition but also as a systemic inflammatory disorder with potential metabolic and immunological consequences. This paradigm shift underscores the need for integrating immunological biomarkers into clinical assessment and therapeutic stratification. Therefore, a critical evaluation of currently available evidence is necessary not only to summarize existing findings but also to identify knowledge gaps and define directions for future research.

## 2. Materials and Methods

This systematic review was conducted in accordance with the PRISMA 2020 guidelines (Table S1). No specific software was used for PRISMA 2020 reporting. A literature search was performed in PubMed, Embase, and Scopus, with Google Scholar used as a supplementary source. The search included English-language publications from January 2015 to June 2026. The following keywords were used in combination: “cytokines”, “serum”, “hidradenitis suppurativa”, and “markers”. In addition, the reference lists and citations of eligible full-text articles were manually screened to identify further relevant studies. Eligible studies for inclusion were original, peer-reviewed human studies reporting serum cytokine levels in patients diagnosed with HS. Reviews, meta-analyses, case reports, editorials, letters, in vitro and animal studies, conference abstracts without the full text available, thematically unrelated publications, and studies assessing cytokines exclusively in tissue samples or other non-serum material were excluded. Study selection included screening of titles and abstracts, followed by full-text assessment of potentially eligible articles. No duplicates were identified during the search process. From each eligible study, the extracted data included the first author, year of publication, study population, cytokines assessed, measurement method, direction of serum cytokine changes, statistical significance, and association with HS severity. Due to heterogeneity among studies, the results were presented as a qualitative narrative synthesis and in tabular form; no meta-analysis was performed. This systematic review was registered in PROSPERO under the registration number CRD420251251812. The review protocol is available in the PROSPERO registry.

## 3. Study Selection

**Figure 1:**
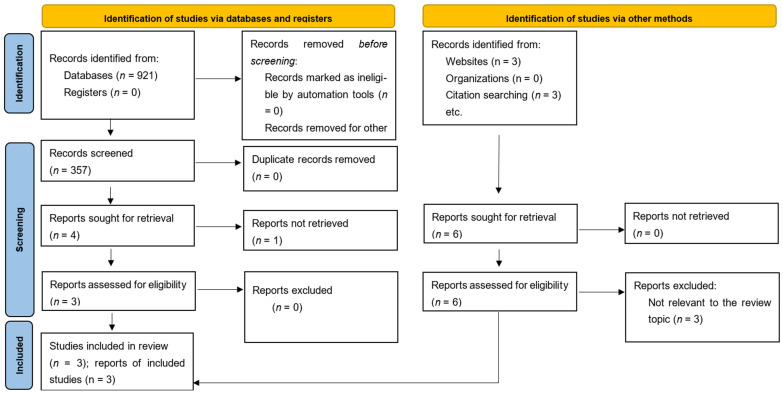
PRISMA flow diagram illustrating the study selection process for the systematic review of serum cytokines in hidradenitis suppurativa. Records excluded after title/abstract screening (*n* = 353); not relevant to the review topic/parameters of interest, *n* = 283 (PubMed: 8; Embase: 2; Scopus: 273); not original research articles, *n* = 70 (PubMed: 1; Embase: 68; Scopus: 1). Source: Page MJ et al. BMJ 2021;372:n71. doi: 10.1136/bmj.n71 [13].

The study selection process is presented in Figure 1. Briefly, the database search identified 12 records in PubMed, 71 in Embase, 838 in Scopus, and three in Google Scholar. After title/abstract screening and full-text eligibility assessment, three PubMed records were included. Records identified in Embase, Scopus, and Google Scholar were excluded because they were not original research articles or were not relevant to the parameters of interest. One potentially relevant Embase record Krueger et al. [12] was excluded because it was available only as a conference abstract and did not meet the eligibility criterion of a full-text original research article. Manual screening of reference lists and citations identified three additional eligible publications. No duplicates were found.

A formal risk-of-bias assessment was not performed, which should be considered a limitation of this review. Owing to substantial heterogeneity among the included studies, no pooled statistical analysis was conducted. The findings were therefore summarized narratively, with attention to the direction of serum cytokine changes, statistical significance as reported by the original authors, and associations with HS severity where available.

The identification and selection process is presented in the PRISMA 2020 flow diagram.

A detailed summary of the selection process, in accordance with PRISMA guidelines, is presented in Figure 1.

## 4. Results

A total of six original studies investigating serum cytokine levels in patients with HS were included in the analysis. The findings consistently demonstrated elevated serum concentrations of various pro-inflammatory cytokines in HS patients compared to healthy controls. Among the most frequently reported cytokines with increased levels were IL-17A, IL-6, IL-1β, IL-23, IL-18, and soluble tumor necrosis factor (TNF) receptors. Despite variations in sample size and methodological approaches across the included studies, these all uniformly indicated heightened systemic inflammatory activity in HS. A summary of the serum-based study findings is presented in Table 1.

**Figure 2:**
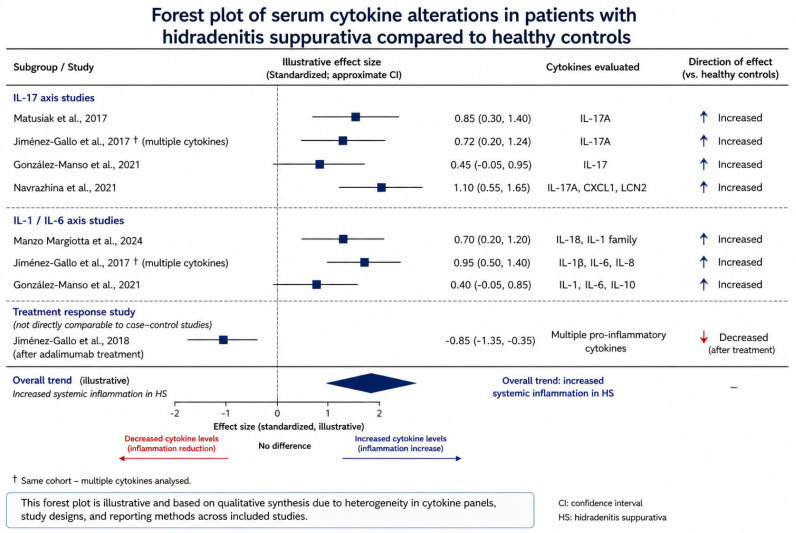
Illustrative forest plot presenting the direction and relative magnitude of changes in serum levels of selected pro-inflammatory cytokines in patients with hidradenitis suppurativa compared to healthy controls [1,2,3,5,14,15].

The results of the included studies are presented as an illustrative forest plot, allowing visual comparison of the direction of cytokine level changes in patients with HS versus healthy controls (Figure 2).

A consistent trend toward increased levels of pro-inflammatory cytokines is observed, particularly within the IL-17 and IL-1/IL-6 axes. Additionally, data from the treatment study indicate a reduction in cytokine levels during biologic therapy, suggesting their potential role as markers of treatment response.

Each study is represented by a point estimate (square) with an approximate confidence interval (horizontal line). The vertical line indicates no difference between groups; values to the right indicate increased cytokine levels in HS patients, while values to the left indicate decreased levels. Subgroup analyses include cytokines related to the IL-17 axis and the IL-1/IL-6 axis. A study evaluating the effect of biologic therapy (adalimumab) is presented separately, as it is not directly comparable to case–control studies. The diamond represents the overall trend across the included studies. This forest plot is illustrative and based on qualitative synthesis due to substantial heterogeneity among the included studies, including differences in cytokine panels, measurement methods, and study designs.

## 5. Discussion

The results of the included studies indicate a consistent involvement of pro-inflammatory cytokines in the pathogenesis of HS [16,17]. Cytokines from the IL-1 and IL-17 families, as well as soluble TNFα receptors, are elevated in HS patients compared to healthy controls, supporting the concept of systemic inflammation. Among these mediators, IL-6, IL-8, IL-17A, IL-18, IL-1β, and soluble TNF receptors appear particularly relevant due to their association with disease activity and severity. These cytokines correlate with clinical severity indices such as IHS4, HS-PGA, Hurley stage, and mHSS, suggesting their potential role as biomarkers of disease burden. Importantly, cytokine levels decrease during biologic therapy [18], especially with adalimumab, and this reduction is associated with clinical improvement. Emerging therapies targeting IL-17, IL-1, and JAK pathways further highlight the central role of cytokine-driven inflammation in HS [19]. Reductions in IL-6, IL-8, CRP, and soluble tumor necrosis factor receptor I levels have also been linked to treatment response.

Elevated levels of IL-1β, IL-6, and IL-17A support the hypothesis that HS is driven by a complex interaction between innate and adaptive immune responses. Transcriptomic data confirm dysregulation of inflammatory pathways, including IL-17 signaling and neutrophil-related chemokines, emphasizing the role of Th17-driven inflammation [20]. The IL-1β–IL-17 axis appears to play a central role in linking follicular damage to chronic immune activation. Neutrophils and Th17 cells further amplify inflammation, contributing to disease persistence and recurrence [21,22]. Current evidence consistently highlights TNF-α and IL-17 as key drivers of inflammation in HS, forming the basis for targeted therapeutic strategies [23]. This is further supported by the observed correlation between cytokine levels and clinical severity indices, suggesting that systemic inflammatory burden reflects disease activity. The inflammatory signature of HS is highly complex and involves multiple immune cell populations and signaling pathways, contributing to its clinical heterogeneity and therapeutic variability [21].

The IL-17 axis deserves special attention, as its relevance has been confirmed in both serum analyses and immunohistochemical assessments of lesional tissue. Patients with more advanced disease show a trend toward higher serum IL-17A levels irrespective of age, gender, BMI, or smoking status, underscoring IL-17A’s potential as a biomarker of disease severity. These findings support the rationale for using biologic agents targeting the IL-17 pathway, which have shown promising early results in clinical settings. The pivotal role of IL-17 in HS pathogenesis is further supported by the clinical efficacy of IL-17 inhibitors, such as secukinumab, which has recently been approved for moderate-to-severe HS [19,21]. Similarly, IL-1 family cytokines, including IL-18 and its regulators, are associated with disease activity [2]. Elevated IL-18 and IL-33 levels further support their role as potential biomarkers of active disease [24].

The potential clinical utility of serum cytokines lies in their ability to serve as minimally invasive biomarkers for disease monitoring and therapeutic response. In particular, dynamic changes in cytokine levels during biologic therapy may provide insight into treatment effectiveness earlier than clinical improvement alone.

However, not all studies have confirmed the utility of cytokines as reliable serum biomarkers, as some reports failed to demonstrate significant differences in IL-17A, IL-1β, or TNF-α levels between HS patients and controls [25]. Importantly, it should be emphasized that no single cytokine has yet demonstrated sufficient specificity to be used as a standalone biomarker. Novel inflammatory indices such as the systemic immune–inflammation index and systemic inflammation response index have also been proposed as promising biomarkers for disease monitoring in HS [26]. A combined biomarker approach integrating cytokines with clinical and metabolic parameters may provide greater diagnostic value.

HS should be considered a systemic inflammatory disease rather than a purely cutaneous condition [15]. The measurement of selected serum cytokines could therefore play both diagnostic and prognostic roles. Inflammatory biomarkers may aid in disease phenotyping, monitoring of therapeutic response, and, importantly, in the era of personalized medicine, facilitate the selection of targeted therapies.

Interpretation of current evidence is limited by small sample sizes and methodological heterogeneity. Differences in patient characteristics, disease severity, and laboratory techniques complicate cross-study comparisons. Additionally, standardization of study design and cytokine measurement is needed in future research.

HS pathogenesis involves not only immune dysregulation but also epithelial and metabolic alterations [25,27].

The IL-1β–IL-17 axis integrates signals from keratinocytes, immune cells, and adipose tissue. Key inflammatory pathways include IL-17 signaling, JAK-STAT signaling, and cytokine–receptor interactions [27].

Importantly, extended biomarker analyses have identified a broad spectrum of molecular targets, with over 100 genes and proteins implicated in HS, many of which are considered “druggable” [27]. This finding highlights the therapeutic potential of targeting key cytokines such as IL-6, IL-17A, and TNF-α, which not only correlate with disease severity but also serve as direct targets of currently available biologic therapies. Clinical improvement observed with TNF-α and IL-17 inhibitors further supports their pathogenic relevance [2,4].

HS is associated with systemic comorbidities, including inflammatory and metabolic disorders [28]. Overlap with pathways involved in atherosclerosis and metabolic regulation supports its systemic nature.

Systemic inflammatory biomarkers such as CRP, erythrocyte sedimentation rate (ESR), and pan-immune–inflammation value (PIV) have been shown to correlate significantly with both disease severity and the presence of metabolic syndrome in patients with hidradenitis suppurativa, highlighting the link between systemic inflammation and clinical burden of the disease [25].

In addition, increasing attention has been given to the role of adipose tissue as an active contributor to systemic inflammation in HS [25]. Adipokines and metabolic mediators may modulate the expression of key cytokines such as IL-6 and TNF-α, thereby influencing both local and systemic inflammatory responses [28]. This may explain the well-documented association between obesity and more severe disease phenotypes.

Given the complexity of these interactions, the use of single cytokines as standalone biomarkers appears insufficient. Instead, a multimarker approach may provide a more accurate reflection of disease activity. The dense network of protein–protein interactions identified in HS suggests a highly integrated inflammatory response, further supporting the need for multidimensional diagnostic and therapeutic strategies [27].

Taken together, the integration of clinical, serological, and molecular data indicates that HS is a highly heterogeneous disease in which cytokines play a central, yet not isolated, role. Their utility as biomarkers may be maximized when combined with the identification of distinct immunological endotypes and supported by multi-omics approaches, representing a promising direction for future research [27,28].

Recent findings suggest that HS can be stratified into two clinicopathogenetic endotypes. These endotypes differ in cytokine profiles, clinical presentation, and potential response to therapy. Applying this subdivision in clinical practice may help identify patients more likely to benefit from specific immune-targeted treatments. Endotyping HS using clinical and serological data could thus serve as the foundation for personalized treatment strategies, with markers such as IL-17, CRP, and lesion localization acting as potential predictors of biologic therapy response.

It is noteworthy that there is significant heterogeneity [29] in Th17-associated immune pathways among HS patients, distinguishing the disease from other inflammatory dermatoses. IL-17 production in HS may be driven by multiple molecular mechanisms, including IL-1β/IL-6, particularly in women, and IL-23, more commonly seen in men or those with lower BMI and less adipose tissue. These pathways may also be hormonally modulated by factors such as estradiol or testosterone, potentially explaining observed gender differences in immunophenotype. This further supports the need to tailor biologic treatments in HS to patient-specific variables such as gender, BMI, immune profile, and hormonal status. For instance, IL-23 inhibitors may be more effective in men with a non-classical monocyte-driven phenotype, while IL-1 or IL-6 inhibitors may be more appropriate for female patients with IL-1β/IL-6 axis predominance.

Proteomic studies confirm a strong neutrophil-driven inflammatory signature in HS [28]. Lipocalin-2 appears to be a promising biomarker associated with disease severity and tunnel formation [30].

Further research is needed to validate cytokines as reliable clinical biomarkers in HS. Future studies should include larger, well-characterized cohorts, standardized methodologies, and the integration of multi-omics approaches to better define immunological endotypes [27,29].

Additionally, investigating the interaction between systemic inflammation and metabolic comorbidities may provide further insight into disease progression and therapeutic outcomes [30,31,32].

## 6. Limitations

This review has several limitations. The number of included studies is limited and mostly involve small sample sizes. Substantial heterogeneity in study populations, disease severity, treatment status, and laboratory methods limit comparability. Due to this variability, quantitative synthesis was not feasible, while the forest plot was illustrative and based on qualitative analysis. Additionally, potential confounders, such as comorbidities, were not consistently reported.

## 7. Conclusions

Hidradenitis suppurativa is increasingly recognized as a systemic inflammatory disease in which pro-inflammatory cytokines play a central role in pathogenesis and disease activity. Elevated serum cytokine levels, their association with clinical severity, and their responsiveness to biologic therapy support their potential utility as biomarkers of disease activity and treatment response. The IL-1β–IL-17 axis appears to be particularly relevant and may represent a promising therapeutic target. At the same time, the immunological heterogeneity of HS suggests that cytokine-based endotyping could support more precise, individualized treatment strategies. However, due to methodological variability and limited available evidence, further validation in larger, well-designed prospective studies is required before routine clinical implementation.

## Figures and Tables

**Table 1:** Overview of serum cytokine profiles in patients with hidradenitis suppurativa.

No.	Author(s) Year	Study Population (HS/Controls)	Cytokines Evaluated	Cytokine Measurement Method	Reported Serum Cytokine Alterations	Statistical Significance	Disease Severity Association
**1**	Matusiak et al. [1], 2017	86/86	IL-17A	ELISA	↑ IL-17A	IL-17A	Associated with Hurley stage and HS-PGA.
**2**	Manzo Margiotta et al. [2], 2024	16/16	IL-1β, IL-18, IL-33, IL-36, IL-1Ra, IL-18BP	Most cytokines, Luminex (multiplex); IL-36α, ELISA; free IL-18, calculated (law of mass action)	↑ IL-18, free IL-18, IL-1Ra, IL-1R2	IL-18, free IL-18	Correlated with severity—IHS4.
**3**	Jiménez-Gallo et al. [3], 2017	74/22	IL-1β, IL-6, IL-8, IL-10, IL-12p70, IL-17A, IL-22, IL-23	xMAP (multiplex bead-based) technology	↑ IL-1β, IL-6, IL-8, IL-10, IL-12p70, IL-17A, sTNF-RII	IL-6, IL-23, sTNF-RI	Correlated with severity—Hurley stage and HS-PGA; ↑ IL-6, IL-8, sTNF-RI, sTNF-RII in more advanced stages of hidradenitis suppurativa.
**4**	Jiménez-Gallo et al [5], 2018	19/19	IL-6, IL-8, IL-10, IL-17A, sTNF-RI, sTNF-RII	xMAP (multiplex bead-based) technology	↓ IL-1β, IL-6, IL-8, IL-10, IL-17A, sTNF-RI, sTNF-RII	IL-6, IL-8, IL-10, IL-17A, sTNF-RII	Reduced cytokines after adalimumab treatment; ↓ IL-6, IL-8, sTNF-RI significantly correlated with clinical improvement.
**5**	Anxo González-Manso et al. [14], 2021	103 patients with HS	IL-1, IL-6, IL-17, IL-10	Not reported	↑ IL-1, IL-6, IL-17, IL-10	IL-10 for cluster 1; IL-1, IL-6, IL-17 for cluster 2	Division into two endotypes: 1—(64.9%) non-obese males, nodular lesions in posterior sites, early onset of HS, higher serum IL-10 levels; 2—(35.1%) obese females or males, lesions in anterior sites, greater presence of sinus tracts and abscesses, fewer nodules, later onset of HS, higher serum levels of IL-1, CRP, IL-17, IL-6.
**6**	Navrazhina K et al. [15], 2021	22/9	IL-17A, CXCL1, Cathepsin D, LCN2, IL-17D, TNF-α, IL-6, ST2, MMP-2, CXCL8 (large-scale proteomic panel of 368 biomarkers)	Multiplex proteomic assay (Olink, PEA technology)	↑ IL-17A, CXCL1, Cathepsin D, LCN2 (particularly in patients with tunnels) ↑ neutrophil-related proteins ↑ ST2 (cardiovascular risk biomarker), altered IL-17D levels	IL-17A, IL-17D	Positive correlation of IL-17A and LCN2 with disease activity (IHS4); higher LCN2 levels in patients with dermal tunnels; increased neutrophil-related proteins in patients with draining tunnels; association with disease severity and cardiovascular risk markers.

Abbreviations: HS—hidradenitis suppurativa; IL—interleukin; IL-1Ra—interleukin-1 receptor antagonist; IL-18BP—interleukin-18 binding protein; IL-1R2—interleukin-1 receptor type 2; TNF-α—tumor necrosis factor alpha; sTNF-RI/sTNF-RII—soluble tumor necrosis factor receptors I and II; CXCL—C-X-C motif chemokine ligand; LCN2—lipocalin-2; MMP-2—matrix metalloproteinase-2; ST2—suppression of tumorigenicity 2 (IL-33 receptor); CRP—C-reactive protein; ELISA—enzyme-linked immunosorbent assay; Luminex—multiplex bead-based immunoassay; xMAP—multi-analyte profiling technology; PEA—proximity extension assay; HS-PGA—Hidradenitis Suppurativa Physician’s Global Assessment; IHS4—International Hidradenitis Suppurativa Severity Score System. Cluster analysis, not HS vs. control comparison. ↑—increase, ↓—decrease.

## Data Availability

Data extracted from the publications included in this review are presented in the manuscript. No analytical code was generated, as no meta-analysis or pooled statistical analysis was performed.

## Supplementary Materials

The following supporting information can be downloaded at: https://www.mdpi.com/article/10.3390/jcm15124699/s1. Table S1: The PRISMA 2020 checklist.
